# Incidence of Cancer and Cardiovascular Disease After Bariatric Surgery in Older Patients

**DOI:** 10.1001/jamanetworkopen.2024.27457

**Published:** 2024-08-13

**Authors:** Peter Gerber, David Naqqar, My von Euler-Chelpin, Joonas H. Kauppila, Giola Santoni, Dag Holmberg

**Affiliations:** 1 Department of Surgery, Capio St Göran’s Hospital, Stockholm, Sweden; 2 Department of Molecular Medicine and Surgery, Karolinska Institutet, and Karolinska University Hospital, Stockholm, Sweden; 3 Department of Public Health, University of Copenhagen, Copenhagen, Denmark; 4 Department of Surgery, Oulu University Hospital and University of Oulu, Oulu, Finland

## Abstract

**Importance:**

Bariatric surgery is associated with decreased risk of obesity-related cancer and cardiovascular disease but is typically reserved for patients younger than 60 years. Whether these associations hold for patients who undergo surgery at older ages is uncertain.

**Objective:**

To determine whether bariatric surgery is associated with a decreased risk of obesity-related cancer and cardiovascular disease in patients who underwent surgery at age 60 years or older.

**Design, Setting, and Participants:**

Population-based cohort study of patients from Denmark, Finland, and Sweden who underwent bariatric surgery at age 60 years or older without previous malignant neoplasm or cardiovascular disease between 1989 and 2019. Each patient who underwent surgery was exactly matched to 5 patients with nonoperative treatment for obesity of the same country, sex, and age at the date of surgery. Data were analyzed in December 2023.

**Exposure:**

Receiving treatment for obesity, including bariatric surgery and nonoperative treatments.

**Main Outcomes and Measures:**

The main outcome was obesity-related cancer, defined as a composite outcome of breast, endometrial, esophageal, colorectal, and kidney cancer, identified from the national cancer registries. The secondary outcome was cardiovascular disease, defined as a composite outcome of myocardial infarction, ischemic stroke, and cerebral hemorrhage, identified from the patient registries. Multivariable Cox regression provided hazard ratios (HR) with 95% CIs adjusted for diabetes, hypertension, peripheral vascular disease, chronic obstructive pulmonary disease, kidney disease, and frailty.

**Results:**

In total, 15 300 patients (median [IQR] age, 63 [61-65] years; 10 152 female patients [66.4%]) were included, of which 2550 (16.7%) had bariatric surgery at age 60 or older and 12 750 (83.3%) had nonoperative treatment. During a median (IQR) of 5.8 (2.8-8.5) person-years of follow-up, 658 (4.3%) developed obesity-related cancer and 1436 (9.4%) developed cardiovascular disease. The risk of obesity-related cancer (HR, 0.81; 95% CI, 0.64-1.03) and cardiovascular disease (HR, 0.86; 95% CI, 0.74-1.01) were similar among who underwent surgery and those who did not. Gastric bypass (1930 patients) was associated with a decreased risk of obesity-related cancer (71 patients [3.7%]; HR, 0.74; 95% CI, 0.56-0.97) and cardiovascular disease (159 patients [8.2%]; HR, 0.82; 95% CI, 0.69-0.99) compared with matched controls (9650 patients; obesity-related cancer: 442 patients [4.6%]; cardiovascular disease: 859 patients [8.9%]).

**Conclusions and Relevance:**

This cohort study found that bariatric surgery in older patients is not associated with lower rates of obesity-related cancer and cardiovascular events, but there was evidence that gastric bypass may be associated with lower risk of both outcomes.

## Key Points

**Question:**

Is bariatric surgery associated with lower rates of obesity-related cancer and cardiovascular disease when performed in patients aged 60 years or older?

**Findings:**

In a cohort study of 2550 patients who underwent bariatric surgery at age 60 years or older, the risk of both obesity-related cancer and cardiovascular disease were similar compared with 12 750 matched controls with nonoperative treatment for obesity.

**Meaning:**

These findings suggest the preventive association between bariatric surgery and obesity-related cancer and cardiovascular disease may be limited to younger individuals.

## Teaser

This cohort study evaluates whether bariatric surgery is associated with a decreased risk of obesity-related cancer and cardiovascular disease in patients who underwent surgery at age 60 years or older.

## Introduction

More than 1 billion people worldwide have obesity, defined as a body mass index of 30 or greater (calculated as weight in kilograms divided by height in meters squared).^1^ Obesity increases the risk of several neoplasms, including cancer of the breast, endometrium, esophagus, colon, rectum, and kidney.^2^ Furthermore, obesity contributes to cardiovascular disease, including hypertension and coronary artery disease, but also comorbidities such as diabetes, osteoarthritis, sleep disorders, and psychiatric disorders.^3^ The most well-documented treatment for obesity is bariatric surgery, which induces rapid, profound, and sustained weight loss in contrast to nonoperative treatment, which includes changes in lifestyle, diet, and physical activity.^4^

Bariatric surgery is associated with a decreased risk of both obesity-related cancer and cardiovascular disease and a prolonged life expectancy in individuals with obesity.^4,5,6,7^ There is controversy regarding whether patients aged older than 60 years should undergo bariatric surgery, as the benefits of bariatric surgery, including weight loss and resolution of obesity-related comorbidities, seem to attenuate with older age.^8^ It is therefore possible that bariatric surgery does not prevent obesity-related cancer and cardiovascular disease in older patients, but data in the literature are scarce. Using nationwide data from 3 countries, we aimed to compare the incidence of obesity-related cancer and cardiovascular disease in patients who had undergone bariatric surgery at age 60 years or older with patients who received nonoperative treatment for obesity.

## Methods

### Design

This was a population-based matched cohort study including nationwide data from Denmark, Finland, and Sweden. The overall study period was from January 1, 1989, until December 31, 2019, but varied across the participating countries (Finland, January 1, 1989, to December 31, 2018; Denmark, July 1, 1996, to December 31, 2018; Sweden, January 1, 1989, to December 31, 2019). Ethical approvals for the study was granted by the regional ethical review board in Stockholm, and data retrieval permissions came from The National Board of Health and Welfare in Sweden, from The Danish Data Protection Agency in Denmark, and from The National Institute for Health and Welfare and Statistics Finland in Finland. Informed consent was waived due to the registry-based design, which only included deidentified data collected from routine health care. The study followed the Strengthening the Reporting of Observational Studies in Epidemiology (STROBE) reporting guideline for cohort studies.^9^

The study population was identified from the Nordic Obesity Surgery Cohort which contains data from the patient registries, cancer registries, and causes of death registries for the 3 countries.^10^ The patient registries have been thoroughly validated for use in epidemiological research and encompass every aspect of health care in the countries, including diagnoses, procedures, hospital stays, outpatient visits, and trips to emergency departments, but exclude primary care.^11,12,13^ The Swedish patient registry has been specifically validated for bariatric surgery with greater than 97% concordance to medical records.^10^

From the patient registries, we identified all patients who underwent bariatric surgery (including gastric bypass, sleeve gastrectomy, gastric banding, and duodenal shunt with biliopancreatic diversion; procedure codes are listed in eTable 1 in Supplement 1) at age 60 years or older. For each patient who underwent surgery, exactly 5 patients with an obesity diagnosis who did not undergo surgery (eTable 2 in Supplement 1) of the same country, sex, and age in years at the date of surgery were selected randomly. Patients with a previous diagnosis of cancer or cardiovascular disease were excluded from the study (eTable 3 in Supplement 1). Start of follow-up occurred on the date of bariatric surgery with patients who did not undergo surgery entering on the same date as their matched patient.

### End Points

The main outcome was obesity-related cancer, defined as a composite outcome including any cancer of the breast, endometrium, esophagus, colon, rectum, and kidney (eTable 4 in Supplement 1). The cancer diagnoses were identified from the national cancer registries, which collect data regarding all malignant tumors diagnosed in the participating countries. The cancer registries of Denmark, Finland, and Sweden have been validated with high completeness and accuracy.^10,14,15^

The secondary outcome was cardiovascular disease, defined as a composite outcome of myocardial infarction, ischemic stroke, and cerebral hemorrhage (eTable 4 in Supplement 1), identified from the patient registries. The Danish and Swedish patient registries have been specifically validated for cardiovascular disease with excellent results.^11,16^ Follow-up ended on the date of diagnosis of obesity-related cancer (for the main outcome only), cardiovascular disease (for the secondary outcome only), bariatric surgery (for patients who did not undergo surgery only), death, or the end of the study period, whichever occurred first.

### Confounders

We considered the following variables as confounders: diabetes, hypertension, peripheral vascular disease, chronic obstructive pulmonary disease, and kidney disease (eTable 5 in Supplement 1). Additionally, we approximated frailty by adjusting for 3 additional variables: deep vein thrombosis or pulmonary embolism, pneumonia, and number of hospital admissions before cohort entry (eTable 5 in Supplement 1). The frailty variables were included to describe and adjust for any apparent selection of more fit candidates for bariatric surgery compared with nonoperative management. Data on all confounders were retrieved from the national patient registries and indexed at study entry.

### Statistical Analysis

Multivariable Cox regression analysis provided hazard ratios (HR) with 95% CIs for both outcomes, adjusted for diabetes (yes or no), hypertension (yes or no), peripheral vascular disease (yes or no), chronic obstructive pulmonary disease (yes or no), kidney disease (yes or no), deep vein thrombosis or pulmonary embolism (yes or no), pneumonia (yes or no), and number of hospital admissions before cohort entry (continuous). SEs were computed using a sandwich estimator clustered on the matching ID variable. The main analysis was conducted with a break-off at exactly 1 year after study entry to allow for latency of the outcomes of bariatric surgery and to avoid biasing the results due to selection of more fit candidates for surgery. Such selection could be declining patients with poor cardiovascular health surgery, optimizing cardiovascular health before surgery, or using diagnostic modalities such as computed tomography or endoscopy to ensure that the patients were free of disease at baseline. All reported HRs were therefore based on person-time and cases occurring after the first year of follow-up unless otherwise stated. Stratified analyses were conducted for the variables country (Denmark, Finland, or Sweden), age (60-65, 66-70, or >70 years), sex (men or women), diabetes (yes or no), and duration of follow-up (<1 year, 1-5 years, 6-10 years, 11- 15 years, or ≥16 years). In a sensitivity analysis, subhazard ratios (SHR) of time to main and secondary outcomes were computed considering time to death a competing event. Finally, a subgroup analysis in patients who underwent gastric bypass surgery, which is the bariatric procedure associated with most weight loss and best metabolic control, and corresponding matched controls was conducted. The proportionality assumption of the Cox models was assessed by computing the Schoenfeld residuals and all analyses satisfied the assumption (eTable 6 in Supplement 1). All analyses were determined in a detailed study protocol completed before data analysis (eAppendix in Supplement 1). The data management and statistical analyses were conducted by a senior biostatistician (G.S.) using the statistical software Stata/MP version 15.1 (StataCorp). Data were analyzed in December 2023.

## Results

### Patients

**Figure 1.**
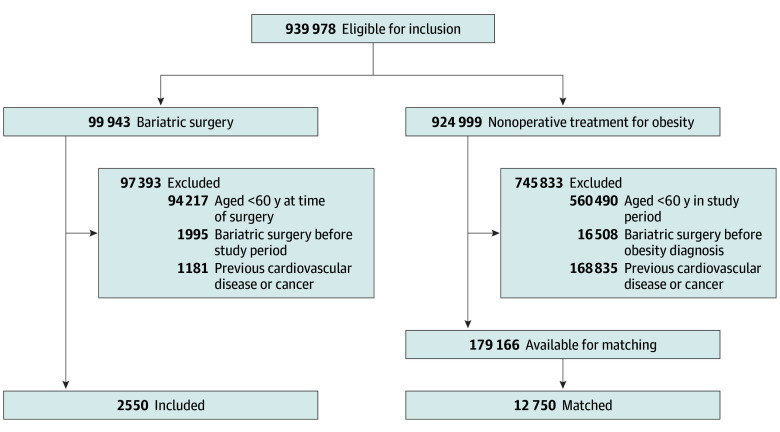
Selection of the Study Patients

In total 15 300 patients were included (median [IQR] age, 63 [61-65] years; 10 152 women [66.4%]), among which 2550 patients (16.7%) had bariatric surgery and 12 750 (83.3%) were matched controls with nonoperative treatment for obesity (Figure 1, Table 1). Patients who underwent surgery were more frequently diagnosed with diabetes and hypertension, but other comorbidities were comparable between the groups at study entry (Table 1).

**Table 1.** Characteristics Among Older Patients With Bariatric Surgery and Matched Controls With Nonoperative Treatment for Obesity

Characteristic	Patients, No. (%)	
	Nonoperative treatment (n = 12 750)	Bariatric surgery (n = 2550)
Sex		
Female	8460 (66.4)	1692 (66.4)
Male	4290 (33.6)	858 (33.6)
Age, median (IQR), y	63 (61-65)	63 (61-65)
60-65	10433 (81.8)	2088 (81.9)
65-70	1955 (15.3)	389 (15.3)
>70	362 (2.8)	73 (2.9)
Year of entry, median (IQR)	2012 (2010-2016)	2012 (2010-2016)
Country		
Denmark	1680 (13.2)	336 (13.2)
Finland	3100 (24.3)	620 (24.3)
Sweden	7970 (62.5)	1594 (62.5)
Diabetes	2325 (18.2)	904 (35.5)
Hypertension	3367 (26.4)	1322 (51.8)
Peripheral vascular disease	183 (1.4)	29 (1.1)
Chronic obstructive pulmonary disease	543 (4.3)	89 (3.5)
Kidney disease	306 (2.4)	41 (1.6)
Frailty		
Deep vein thrombosis or pulmonary embolism	130 (1.0)	21 (0.8)
Pneumonia	272 (2.1)	40 (1.6)
Previous hospital admissions, median (IQR), No.	20 (11-37)	22 (12-38)
90-d mortality	41 (0.32)	8 (0.31)
Obesity-related cancer	557 (4.4)	101 (4.0)
Follow-up time, median (IQR), y	5.8 (2.8-8.5)	6.1 (3.0-8.7)
Cardiovascular disease	1212 (9.5)	224 (8.8)
Follow-up time, median (IQR), y	5.5 (2.6-8.3)	5.8 (2.9-8.5)
Myocardial infarction	536 (4.2)	81 (3.2)
Cerebrovascular disease	760 (6.0)	155 (6.1)

### Obesity-Related Cancer

**Figure 2.**
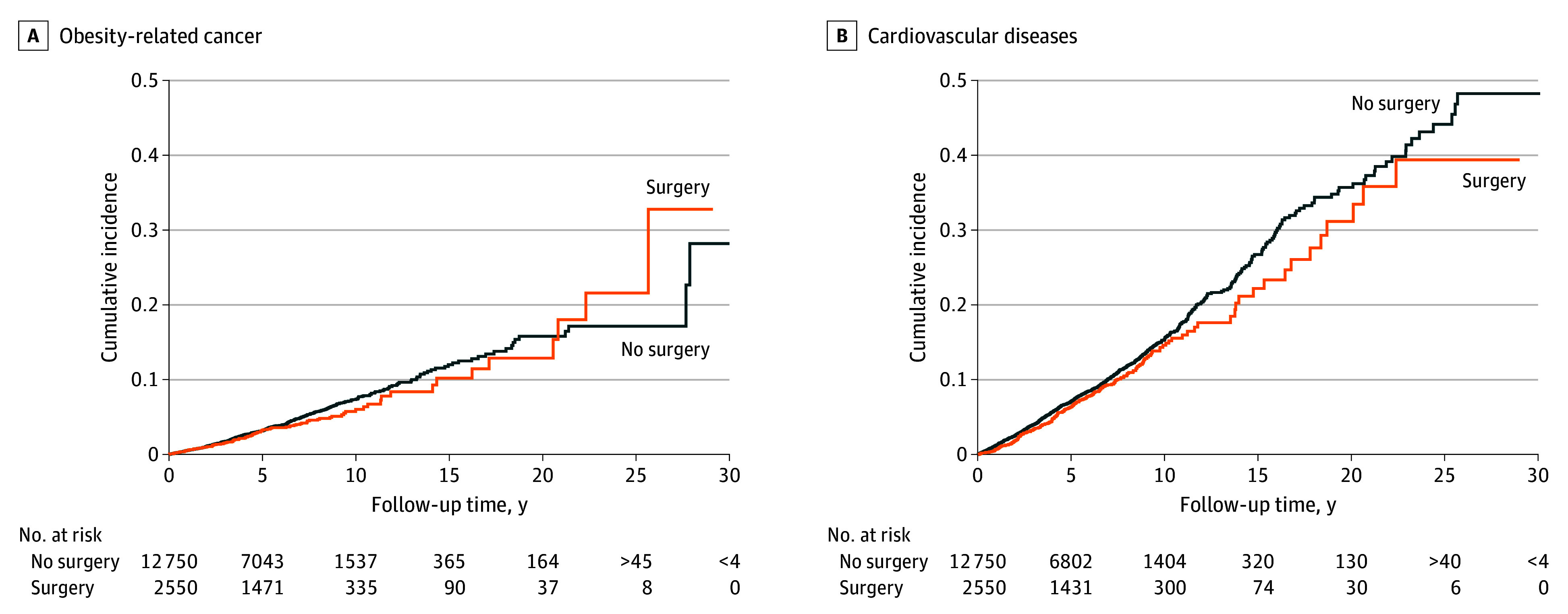
Cumulative Incidence of Obesity-Related Cancer A, Cumulative incidence of obesity-related cancer among older patients with bariatric surgery compared with matched controls. B, Cumulative incidence of cardiovascular disease among older patients with bariatric surgery compared with matched controls.

During a median (IQR) 5.8 (2.8-8.5) years of follow-up, 101 patients with bariatric surgery (4.9%) and 557 patients with nonoperative treatment (4.4%) developed obesity-related cancer (Table 1, Figure 2A). The overall risk of obesity-related cancer was similar among patients with bariatric surgery compared with those with nonoperative treatment (adjusted HR, 0.81; 95% CI, 0.64-1.03) (Table 2). Stratified analyses demonstrated a 24% decreased risk of obesity-related cancer in women who underwent bariatric surgery (HR, 0.76; 95% CI, 0.58-0.99), but there was otherwise no clear benefit of surgery (Table 2). The risk of obesity-related cancer was similarly comparable in patients who underwent surgery and those who did not in a sensitivity analysis accounting for mortality as a competing risk (SHR, 0.85; 95% CI, 0.67-1.07). Among 1930 patients who underwent gastric bypass, 71 (3.7%) developed obesity-related cancer, in comparison with 442 in 9650 matched patients (4.6%) with nonoperative treatment. After adjustments, gastric bypass was associated with a 26% decreased risk for obesity-related cancer (HR, 0.74; 95% CI, 0.56-0.97) (eTable 7 and eTable 8 in Supplement 1).

**Table 2.** Risk of Obesity-Related Cancer in Older Patients Treated With Bariatric Surgery vs Nonoperative Treatment for Obesity

Characteristic	Nonoperative treatment		Bariatric surgery			
	Person-years	Cases, No.	Person-years	Cases, No.	HR (95% CI)^a^	
					Unadjusted	Adjusted
Total^b^	64 152	490	13 486	88	0.85 (0.68-1.07)	0.81 (0.64-1.03)
Follow-up period						
≤1 y	12 100	67	2436	13	0.96 (0.54-1.74)	1.07 (0.58-1.98)
1-5 y	43 608	295	9032	56	0.92 (0.69-1.22)	0.85 (0.65-1.17)
6-10 y	16 377	156	3515	21	0.63 (0.39-1.00)	0.60 (0.37-0.98)
11-15	2629	26	598	5	0.85 (0.32-2.28)	0.79 (0.26-2.41)
≥16	1537	13	341	6	2.00 (0.73-5.44)	1.92 (0.71-5.23)
Sex^b^						
Male	20 428	96	4320	21	1.03 (0.64-1.66)	0.96 (0.60-1.55)
Female	43 724	394	9166	67	0.81 (0.62-1.05)	0.76 (0.58-0.99)
Age, y^b^						
60-65	53 584	400	11 321	74	0.87 (0.68-1.12)	0.87 (0.68-1.12)
65-70	8928	77	1891	>10	0.73 (0.39-1.37)	0.70 (0.38-1.31)
>70	1640	13	274	<4	0.95 (0.23-3.91)	0.94 (0.23-3.87)
Country^b^						
Sweden	42 424	342	9005	58	0.79 (0.60-1.05)	0.83 (0.64-1.08)
Finland	12 730	75	2621	16	1.04 (0.60-1.78)	0.97 (0.56-1.68)
Denmark	8998	73	1861	14	0.93 (0.51-1.68)	0.90 (0.50-1.63)
Diabetes^b^						
No	53 448	399	8897	59	0.88 (0.67-1.16)	0.86 (0.65-1.13)
Yes	10 703	91	4590	29	0.74 (0.48-1.12)	0.72 (0.47-1.10)

Abbreviation: HR, hazard ratio.

^a^ Patients who did not undergo surgery served as the reference group.

^b^ Excluding the first year of follow-up.

### Cardiovascular Disease

During a median (IQR) 5.6 (2.6-8.3) years of follow-up, 224 patients with bariatric surgery (8.8%) developed cardiovascular disease compared with 1212 patients with nonoperative treatment (9.5%) (Table 1, Figure 2B). The overall risk of cardiovascular disease was similar among patients with bariatric surgery and nonoperative treatments (HR, 0.86; 95% CI, 0.74-1.01) (Table 3). Patients with bariatric surgery had a 45% decreased risk of cardiovascular disease in the first year after surgery (HR, 0.55; 95% CI, 0.33-0.91). Stratified analyses revealed a 17% decreased risk of cardiovascular disease among patients who underwent surgery at age 60 to 65 years (HR, 0.83; 95% CI, 0.70-0.99), but there were otherwise no clear benefits of bariatric surgery (Table 3). In a sensitivity analysis accounting for mortality as a competing risk, patients who underwent surgery had a similar risk of cardiovascular disease as the patients who did not undergo surgery (SHR, 0.90; 95% CI, 0.78-1.05). In total, 159 patients (8.2%) with gastric bypass developed cardiovascular disease, in comparison with 859 (8.9%) with nonoperative treatment. After adjustments, gastric bypass was associated with a 18% decreased risk of cardiovascular disease compared with patients with nonoperative treatment (HR, 0.82; 95% CI, 0.69-0.99) (eTable 7 and eTable 8 in Supplement 1).

**Table 3.** Risk of Cardiovascular Disease in Older Patients Treated With Bariatric Surgery vs Nonoperative Treatment for Obesity

Characteristic	Nonoperative treatment		Bariatric surgery			
	Person-years	Cases, No.	Person-years	Cases, No.	HR (95% CI)^a^	
					Unadjusted	Adjusted
Total^b^	61 676	1059	12 946	207	0.93 (0.80-1.08)	0.86 (0.74-1.01)
Follow-up						
≤1 y	12 065	153	2434	17	0.55 (0.33-0.91)	0.55 (0.33-0.91)
1-5 y	42 618	648	8859	131	0.97 (0.81-1.17)	0.90 (0.74-1.09)
6-10 y	15 492	307	3300	59	0.90 (0.68-1.19)	0.84 (0.63-1.11)
11-15	2321	72	511	9	0.68 (0.29-1.12)	0.54 (0.27-1.09)
≥16	1245	32	276	8	1.14 (0.50-2.59)	1.17 (0.52-2.59)
Sex^b^						
Male	19 201	404	4113	78	0.90 (0.70-1.14)	0.82 (0.64-1.04)
Female	42 474	655	8834	129	0.94 (0.78-1.14)	0.90 (0.74-1.09)
Age, y^b^						
60-65	51 653	830	10 932	158	0.90 (0.75-1.06)	0.83 (0.70-0.99)
65-70	8507	176	1781	39	1.05 (0.76-1.46)	0.99 (0.71-1.37)
>70	1516	53	234	10	1.33 (0.66-2.67)	1.31 (0.66-2.62)
Country^b^						
Sweden	40 786	723	8620	143	0.93 (0.78-1.11)	0.88 (0.73-1.05)
Finland	12 106	210	2492	47	1.09 (0.80-1.49)	0.98 (0.72-1.34)
Denmark	8783	126	1834	17	0.65 (0.39-1.08)	0.61 (0.36-1.02)
Diabetes^b^						
No	51 516	814	8567	122	0.89 (0.74-1.08)	0.89 (0.73-1.07)
Yes	10 159	245	4379	85	0.80 (0.63-1.02)	0.83 (0.65-1.06)

Abbreviation: HR, hazard ratio.

^a^ Patients who did not undergo surgery served as the reference group.

^b^ Excluding the first year of follow-up.

## Discussion

The main finding of this study was that bariatric surgery in older patients was not associated with a decreased risk of obesity-related cancer or cardiovascular disease. It appeared to be associated with a limited benefit in certain subgroups, however, such as a decreased risk of obesity-related cancer in women and a decreased risk of both obesity-related cancer and cardiovascular disease in patients who underwent gastric bypass.

The long-term risks for obesity-related cancer and cardiovascular disease postbariatric surgery have been studied extensively in adults younger than 60 years. A systematic review and meta-analysis^17^ of 33 cohort studies including a total of 25 632 528 patients showed that those who underwent bariatric surgery had a 44% reduced risk of obesity-related cancer (OR, 0.56; 95% CI, 0.46-0.68). Another systematic review and meta-analysis^6^ of 39 cohort studies indicated a reduced risk of several cardiovascular diseases, such as myocardial infarction (HR, 0.58; 95% CI, 0.43-0.76), stroke (HR, 0.64; 95% CI, 0.53-0.77), and cardiovascular mortality (HR, 0.59; 95% CI, 0.47-0.73) following bariatric surgery compared with conservative nonoperative treatment. However, these studies were conducted in younger patients and the association between bariatric surgery and obesity-related cancer or cardiovascular disease specifically in patients older than 60 years was not investigated.

Some previous studies have examined the resolution of comorbidities in older patients undergoing bariatric surgery. A large Swedish cohort study^8^ of 2687 patients undergoing gastric bypass at age 60 years or older found inferior resolution of diabetes (OR, 0.70; 95% CI, 0.57-0.86) and hypertension (OR, 0.45; 95% CI, 0.37-0.53) compared with those who had gastric bypass before age 60 years. A single-center cohort study in 83 patients with bariatric surgery at age 60 years or older found a similar improvement in obesity-related comorbidities as in younger patients who underwent surgery but was probably underpowered to find smaller differences in comorbidity improvement.^18^ Another single-center cohort study of 500 patients undergoing bariatric surgery found that patients who underwent surgery at age 60 years or older had less weight loss and lower resolution of comorbidities compared with younger patients who underwent surgery.^19^ These studies show that bariatric surgery is slightly less efficient with respect to weight loss and comorbidity resolution when performed in older patients.

In this study, older patients with bariatric surgery did not have a decreased risk of obesity-related cancer or cardiovascular disease. These findings may be explained by the poorer weight loss and resolution of comorbidities observed in patients who underwent surgery at an older age.^8,17,18^ However, we found that women had a lower risk for obesity-related cancer after bariatric surgery. This might be explained by the fact that weight loss reduces the circulating levels of estrogen, which could consequently reduce the risk of estrogen-sensitive cancers such as endometrial and breast cancer.^20^ The risk of cardiovascular disease was clearly reduced within 1 year of surgery, but the association attenuated to null after this initial period. The initial protective association may be explained by the catabolic state induced by bariatric surgery as a possible protection against cardiovascular diseases.^21^ Additionally, patients who underwent gastric bypass had a slight but significantly lower risk for both obesity-related cancer and cardiovascular disease. Gastric bypass induces profound weight loss and has a highly effective metabolic impact, which may explain a more powerful association in contrast to all bariatric procedures.^22^ Similarly, gastric bypass appears to be the most powerful bariatric procedure in the prevention of obesity-related cancer and cardiovascular disease in younger patients.^23^

### Strengths and Limitations

This was the first study we know of to examine the risk of obesity-related cancer and cardiovascular disease in older patients undergoing bariatric surgery. Methodological strengths of the study included the nationwide coverage of patients from 3 Nordic countries, which allowed for a large sample size of patients followed up for a long period of time. The data sources used in the study have been extensively validated specifically for both cancer diagnoses and cardiovascular disease with excellent results. The large sample size allowed for stratified analyses, which identified subgroups that could benefit from bariatric surgery. Due to the nationwide design with mandatory participation among all permanent residents of the 3 participating countries, follow-up was complete and there were no missing data. The long follow-up time allowed for a long latency between surgery and outcomes and the avoidance of selection bias due to losses to follow-up. Finally, the population-based design facilitated the generalization of the results to countries with similar health care structures and demographics as the participating countries (eg, the other Nordic countries).

This study had limitations. One limitation was potential residual confounding, including missing data on body mass index and smoking, which were not available from the national health registries. We took steps to control confounding by matching and adjustments for comorbidities associated with both obesity-related cancer and cardiovascular disease, thereby indirectly adjusting for any residual confounding as well. After the matching process, we found the group who underwent surgery and the group who did not to be similar with respect to most comorbidities and frailty, indicating no clear differences between the groups. The exceptions were diabetes and hypertension, which was expected, as the presence of these comorbidities increases the indication for having bariatric surgery. Similarly, unobserved selection cannot be excluded, as patients accepted for and accepting to undergo bariatric surgery may be different from those refraining from or being declined surgery (eg, regarding fitness, cardiopulmonary health, or psychiatric stability). Some of these more nuanced differences may represent a systematic difference between patients who undergo surgery and those who do not, which may not be possible to measure using registry data but may only be observable in the clinical setting and may therefore not have been corrected in the matching and adjustment process. Thus, the observational design and possibilities for residual confounding and selection prohibits a causal interpretation of the results.

It should also be acknowledged that the null association between bariatric surgery and outcomes observed in this study may be due to imprecision (ie, limited power). While the sample size of the study was comparably large, both main and subanalyses consistently yielded results with point estimates below 1 but with CIs encompassing 1. It is therefore possible that future studies with larger sample sizes and lower risk of random error will provide statistically significant results supporting bariatric surgery as a method to reduce incidence of obesity-related cancer and cardiovascular disease in older patients. This uncertainty will likely be answered when more data from other study settings are available. Additionally, the results should be generalized to patients currently receiving bariatric surgery and not necessarily to patients receiving bariatric surgery in the future (eg, in the circumstance where the eligibility criteria for bariatric surgery is expanded beyond the age of 60 years).

## Conclusions

In this cohort study of older patients with obesity, bariatric surgery was not associated with a lower risk of obesity-related cancer or cardiovascular disease compared with nonoperative treatment. Women and patients undergoing gastric bypass treatment seemed to benefit from surgery to a lesser extent. The findings from this study suggest a limited role of bariatric surgery in older patients for the prevention of obesity-related cancer or cardiovascular disease.

## Notes

Supplement 1. **eTable 1. **Procedural Codes for Bariatric Surgery **eTable 2. **Diagnosis Codes for Obesity **eTable 3. **Diagnosis Codes for Exclusion Criteria **eTable 4. **Diagnosis Codes for Main and Secondary Outcomes **eTable 5. **Diagnosis Codes for Confounders **eTable 6. ***P*-values for the Null Hypothesis that the Proportionality Assumption of the Cox Regression is Valid (Analysis of the Schoenfeld Residuals on Time) **eTable 7. **Characteristics Among Older Patients With Gastric Bypass and Matched Controls With Nonoperative Treatment For Obesity **eTable 8. **Risk of Obesity-Related Cancer and Cardiovascular Disease in Older Patients With Gastric Bypass Versus Nonoperative Treatment for Obesity **eAppendix. **Study Protocol
eReferences.

Supplement 2.
Data Sharing Statement
