# Supplementary material for: Incidence of Cancer and Cardiovascular Disease After Bariatric Surgery in Older Patients
Source: JAMA Netw Open. 2024 Aug 13;7(8):e2427457. doi: 10.1001/jamanetworkopen.2024.27457 (PMC11322843; doi:10.1001/jamanetworkopen.2024.27457)
Supplement: Supplement 1. — eTable 1. Procedural Codes for Bariatric Surgery eTable 2. Diagnosis Codes for Obesity eTable 3. Diagnosis Codes for Exclusion Criteria eTable 4. Diagnosis Codes for Main and Secondary Outcomes eTable 5. Diagnosis Codes for Confounders eTable 6. P-values for the Null Hypothesis that the Proportionality Assumption of the Cox Regression is Valid (Analysis of the Schoenfeld Residuals on Time) eTable 7. Characteristics Among Older Patients With Gastric Bypass and Matched Controls With Nonoperative Treatment For Obesity eTable 8. Risk of Obesity-Related Cancer and Cardiovascular Disease in Older Patients With Gastric Bypass Versus Nonoperative Treatment for Obesity eAppendix. Study Protocol eReferences. [file jamanetwopen-e2427457-s001.pdf]

Supplemental Online Content

Gerber P, Naqqar D, von Euler-Chelpin M, Kauppila JH, Santoni G, Holmberg D. Incidence of cancer and cardiovascular disease after bariatric surgery in older patients. *JAMA Netw Open*. 2024;7(8):e2427457. doi:10.1001/jamanetworkopen.2024.27457

eTable 1. Procedural Codes for Bariatric Surgery
eTable 2. Diagnosis Codes for Obesity
eTable 3. Diagnosis Codes for Exclusion Criteria
eTable 4. Diagnosis Codes for Main and Secondary Outcomes
eTable 5. Diagnosis Codes for Confounders
eTable 6. P-values for the Null Hypothesis that the Proportionality Assumption of the Cox Regression is Valid (Analysis of the Schoenfeld Residuals on Time)
eTable 7. Characteristics Among Older Patients With Gastric Bypass and Matched Controls With Nonoperative Treatment For Obesity
eTable 8. Risk of Obesity-Related Cancer and Cardiovascular Disease in Older Patients With Gastric Bypass Versus Nonoperative Treatment for Obesity
eAppendix. Study Protocol
eReferences.

This supplemental material has been provided by the authors to give readers additional information about their work.

© 2024 Gerber P et al. *JAMA Network Open.*

**eTable 1. Procedural Codes for Bariatric Surgery**			
**Operation**	**NOMESCO[a] (After 1997)**	**Swedish Classification (Before 1997)**	**Finnish Classification (Before 1997)**
**Vertical-Banded Gastroplasty**		4751	
**Gastric Bypass**	JDF10-11, JDF50-51	4752	
**Gastric Banding**	JDF20-21	4753	
**Sleeve Gastrectomy**	JDF96 (after 2000), JDF97 (after 2000), JDF40-41, JDF00-01 (after 2000)	-	-
**Other**	JDF00-01	4759	6548, 6559
**Duodenal Shunt With Biliopancreatic Diversion**	JFD03-04	4750	
[a]From 1996 in Denmark			

**eTable 2. Diagnosis Codes for Obesity**				
	**ICD-7**	**ICD-8**	**ICD-9**	**ICD-10**
**Any Country**	287	277	278A	E66
**Sweden**			278A, 278B	
**Finland**			2780, 2781	

© 2024 Gerber P et al. *JAMA Network Open.*

eTable 3. Diagnosis Codes for Exclusion Criteria			
	**ICD-8**	**ICD-9**	**ICD-10**
**Country**	Denmark before 1993, Finland and Sweden before 1987	Finland and Sweden 1987-1996	Finland and Sweden 1997-, Denmark 1994-
**Cardiovascular Disease**	410-414	410-414	I20-I25
**Cerebrovascular Disease**	430-438, 344	430–438, 362D, 342-344	G45–G46, I60–I69
**Any Malignancy or Metastasis (Except Non-Melanoma Skin Cancer)**	140-172, 174-209	140–172, 174–208	C00–C43, C45-C97

© 2024 Gerber P et al. *JAMA Network Open.*

eTable 4. Diagnosis Codes for Main and Secondary Outcomes				
	**Obesity-Related Cancer**			
	**Sweden**		**Other Nordic Countries**	
	**ICD-7**	**24.1 Histology code**	**ICD-10**	**ICD-O/3**
**Esophageal Adenocarcinoma**	150	096	C15	8140-8149, 8160-8162, 8190-8221, 8260-8337, 8350-8551, 8570-8576, 8940-8941 (all with fifth digit 3)
**Gastric Cardia Adenocarcinoma**	1511	096	C160	8140-8149, 8160-8162, 8190-8221, 8260-8337, 8350-8551, 8570-8576, 8940-8941 (all with fifth digit 3)
**Endometrial Cancer**	172		C54	
**Colorectal Cancer**	153, 154		C18, C19, C20	
**Breast Cancer**	170		C50	
**Kidney Cancer**	180.0, 180.9, 180.1		C64, C65	
	**Cardiovascular Disease**			
	**ICD-9**		**ICD-10**	
**Myocardial Infarction**	410, 412		I20.0, I21-I24	
**Cerebrovascular Disease**	430-436		G45-G46, I60-I64	

© 2024 Gerber P et al. *JAMA Network Open.*

eTable 5. Diagnosis Codes for Confounders		
	**ICD-9**	**ICD-10**
**Diabetes**	250	E11-E14
**Hypertension**	401-405	I10-I15
**Peripheral Vascular Disease**	440–447, V43E, 785E	I70–I73, I770–I771, K551, K558–K559, R02, Z958–Z959
**Chronic Obstructive Pulmonary Disease, Emphysema, Bronchitis, Bronchiectasis (Smoking-Related Lung Disorders)**	466, 490-492, 494, 496	J40-J41, J43-J44, J47
**Renal Disease**	403– 404, 580–588, V42A, V45B	I12–I13, N01, N03, N05, N07–N08, N171–N172, N18, N19, N25, Z49, Z940, Z992
**Deep Vein Thrombosis or Pulmonary Embolism**	415B, 416W, 451B	I802, I26
**Pneumonia**	480-486	J12-18

© 2024 Gerber P et al. *JAMA Network Open.*

eTable 6. *P*-values for the Null Hypothesis that the Proportionality Assumption of the Cox Regression is Valid (Analysis of the Schoenfeld Residuals on Time)

	**P-Value of the Unadjusted Model**	**P-Value of the Adjusted Model**
**Outcome**[a]		
**Obesity-Related Cancer**		
**Global Test**	0.410	0.501
**Test for Surgery**	0.410	0.406
**Cardiovascular Disease**		
**Global Test**	0.571	0.686
**Test for Surgery**	0.571	0.699

[a]Excluding the first year of follow-up

© 2024 Gerber P et al. *JAMA Network Open.*

**eTable 7. Characteristics Among Older Patients With Gastric Bypass and Matched Controls With Nonoperative Treatment For Obesity**		
**Characteristic**	**Non-Operative Treatment, No. (%)**	**Gastric Bypass, No. (%)**
**Total**	9650 (83.3)	1930 (16.7)
**Sex**		
**Male**	3330 (34.5)	666 (34.5)
**Female**	6320 (65.5)	1264 (65.5)
**Age, median (IQR), y**	63 (61-64)	63 (61-64)
**≤65**	8166 (84.6)	1634 (84.7)
**65-70**	1352 (14.0)	269 (13.9)
**>70**	132 (1.4)	27 (1.4)
**Year of entry, median (IQR)**	2012 (2010-2015)	2012 (2010-2015)
**Country**		
**Denmark**	1390 (14.4)	278 (14.4)
**Finland**	2130 (22.1)	426 (22.1)
**Sweden**	6130 (63.5)	1226 (63.5)
**Diabetes**	1806 (18.8)	737 (38.2)
**Hypertension**	2652 (27.5)	1042 (54.0)
**Peripheral vascular disease**	134 (1.4)	24 (1.2)
**Chronic obstructive pulmonary disease**	410 (4.3)	64 (3.3)
**Renal disease**	222 (2.3)	21 (1.1)
**Frailty**		
**Deep vein thrombosis or pulmonary embolism**	89 (0.9)	17 (0.9)
**Pneumonia**	198 (2.1)	29 (1.5)
**Number of hospital admissions, median (IQR)**	20 (11-36)	22 (12-37)
**90-day mortality**	31 (0.3)	4 (0.2)
**Obesity-related cancer**	442 (4.6)	71 (3.7)
**Follow-up time, median (IQR), y**	6.2 (3.2-8.5)	6.6 (3.7-8.7)
**Cardiovascular disease**	859 (8.9)	159 (8.2)
**Follow-up time, median (IQR), y**	6.0 (3.1-8.3)	6.3 (3.4-8.6)

Abbreviations: IQR – interquartile range, y – years

© 2024 Gerber P et al. *JAMA Network Open.*

eTable 8. Risk of Obesity-Related Cancer and Cardiovascular Disease in Older Patients With Gastric Bypass Versus Nonoperative Treatment for Obesity							
**Obesity-Related Cancer**							
	**Non-Operative Treatment**			**Gastric Bypass**			
**Characteristic**	**Person-years**	**Cases (n)**	**HR**	**Person-years**	**Cases (N)**	**Unadjusted HR (95% CI)**	**Adjusted HR (95% CI)**
**Total[a]**	48848	390	Ref	10275	65	0.79 (0.61-1.03)	0.74 (0.56-0.97)
**Follow-up**							
**≤1 year**	9238	52	Ref	1865	6	0.57 (0.25-1.33)	0.62 (0.26-1.46)
**1-5 years**	35070	247	Ref	7292	50	0.97 (0.72-1.32)	0.91 (0.67-1.25)
**6-10 years**	12569	130	Ref	2704	>10	0.47 (0.26-0.83)	0.44 (0.24-0.80)
**≥11 years**	1209	13	Ref	279	<4	0.69 (0.15-3.24)	0.62 (0.11-3.44)
**Cardiovascular Disease**							
	**Non-Operative Treatment**			**Gastric Bypass**			
	**Person-years**	**Cases (n)**	**HR**	**Person-years**	**Cases (n)**	**Unadjusted HR (95% CI)**	**Adjusted HR (95% CI)**
**Total***	47391	757	Ref	9956	145	0.91 (0.76-1.09)	0.82 (0.69-0.99)
**Follow-up**							
**≤1 year**	9219	102	Ref	1862	14	0.68 (0.39-1.19)	0.62 (0.35-1.10)
**1-5 years**	34357	505	Ref	7169	92	0.87 (0.70-1.09)	0.80 (0.64-1.00)
**6-10 years**	11965	221	Ref	2556	46	0.98 (0.71-1.34)	0.86 (0.62-1.18)
**≥11 years**	1070	31	Ref	231	7	1.06 (0.47-2.40)	0.93 (0.40-2.17)

[a]Excluding the first year of follow-up

Abbreviations: CI – confidence interval; HR – hazard ratio

© 2024 Gerber P et al. *JAMA Network Open.*

eAppendix. Study Protocol

Version: 4.0

Date: 2023-12-08

Preliminary Title: Risk of obesity-related cancer and cardiovascular disease after bariatric surgery in the elderly

Investigators: Peter Gerber (first author), Giola Santoni, My Caterina von Euler-Chelpin, Joonas Kauppila, Dag Holmberg (last author)

Student: David Naqqar

BACKGROUND

Obesity, defined as body mass index (BMI) >30, is an increasing global health problem that currently affects 30-40% of adults in the Western world.[1] Obesity is associated with comorbidities, mainly diabetes, hypertension, cardiovascular diseases, cancer, and psychiatric disorders, as well as reduced overall survival.[2, 3] Severely obese individuals (BMI ≥35) may be selected for bariatric surgery, which induces rapid, pronounced and long-lasting weight loss, and in many cases resolution of obesity-related comorbidities.[4-11]

There is controversy whether patients aged 60 years or older should be recommended for bariatric surgery, as the complication rate may be increased and the weight loss decreased when compared to younger individuals.[12, 13] Few studies have examined the potential long-term benefits of bariatric surgery for obesity in patients aged over 60 years, because studies have generally been small (n<100) and follow-up short (<2 years). Using nationwide data from three Nordic countries, we set out to assess the risk of long-term risk of obesity-related cancer and major cardiovascular events in patients >60 years old when they undergo bariatric surgery as compared to patients >60 years with standard care for obesity.

HYPOTHESIS

The risks of obesity-related cancer and cardiovascular diseases are decreased after obesity surgery in patients 60 years or older compared to matched controls with obesity.

METHODS

Study design: Population-based cohort study in three Nordic countries.

Study period: Limited to the introduction of ICD-9 in the Nordic countries.

- Denmark: July 1, 1996, to December 31, 2018

© 2024 Gerber P et al. *JAMA Network Open.*

- Finland: January 1, 1989, to December 31, 2018
- Sweden: January 1, 1989, to December 31, 2019

Study population: All individuals with a primary bariatric surgery at age >60 years will be identified from the patient registries. These patients will be matched by country, sex, age, and calendar year in a 1:5 ratio to individuals with an obesity diagnosis according to the patient registries. Bariatric surgery and obesity are defined by the codes listed in Table 1 and 2. Patients with a previous diagnosis of cancer or cardio/cerebrovascular disease are excluded from the study population (Table 3).

Table 1. Codes for Bariatric Surgery

Operation	NOMESCO* (1997-)	Old Swedish classification (before 1997)	Old Finnish classification (before 1997)
**Vertical-Banded Gastroplasty**		4751	
**Gastric bypass**	JDF10-11, JDF50-51	4752	
**Gastric banding**	JDF20-21	4753	
**Sleeve gastrectomy**	JDF96 (after 2000), JDF97 (after 2000), JDF40-41, JDF00-01 (after 2000)	-	-
**Other**	JDF00-01	4759	6548, 6559
**Duodenal shunt with biliopancreatic diversion**	JFD03-04	4750	

***From 1996 in Denmark**

Table 2. Obesity codes (icd 7 should not be needed)

	ICD-7	ICD-8	ICD-9	ICD-10
**Any country**	287	277	278A	E66
**Sweden**			278A, 278B	

© 2024 Gerber P et al. *JAMA Network Open.*

**Finland**			2780, 2781	

**Table 3. ICD-codes for exclusion criteria.**			
	**ICD-8**	**ICD 9**	**ICD 10**
**Country**	Denmark before 1993, Finland and Sweden before 1987	Finland and Sweden 1987-1996	Finland and Sweden 1997-, Denmark 1994-
**Cardiovascular disease**	410-414	410-414	I20-I25
**Cerebrovascular disease including transient ischemic attack**	430-438, 344	430–438, 362D, 342-344	G45–G46, I60–I69
**Any malignancy or metastasis (except non-melanoma skin cancer)**	140-172, 174-209	140–172, 174–208	C00–C43, C45-C97

Matching criteria:

- Country: Denmark, Finland, or Sweden
- Age in years: Continuous
- Calendar year: Continuous.
- Sex: Male or female.

Data source: Nordic Obesity Surgery Cohort (NordOSCo), which holds the following registries of interest for this study:

- Patient registries
- Cancer registries
- Cause of death registries
- Scandinavian Obesity Surgery Registry (SOReg) (Sweden only)

© 2024 Gerber P et al. *JAMA Network Open.*

Outcomes:

Main:

- Obesity-related cancer, including esophageal adenocarcinoma (and cardia adenocarcinoma), endometrial, colorectal, breast, and kidney) as a composite outcome. (Table 4)

Secondary:

- Cardiovascular disease, including myocardial infarction, ischemic stroke, and cerebral hemorrhage (Table 4).

Table 4. Definitions of outcomes.

	Obesity-related cancer			
	Sweden		Other Nordic countries	
	**ICD-7**	**24.1 Histology code**	**ICD-10**	**ICD-O/3**
**Esophageal adenocarcinoma**	150	096	C15	8140-8149, 8160-8162, 8190-8221, 8260-8337, 8350-8551, 8570-8576, 8940-8941 (all with fifth digit 3)
**Gastric cardia adenocarcinoma**	1511	096	C160	8140-8149, 8160-8162, 8190-8221, 8260-8337, 8350-8551, 8570-8576, 8940-8941 (all with fifth digit 3)
**Endometrial cancer**	172		C54	
**Colorectal cancer**	153, 154		C18, C19, C20	
**Breast cancer**	170		C50	
**Kidney cancer (including renal pelvis)**	180.0, 180.9, 180.1		C64, C65	
	**Cardiovascular disease**			
	**ICD-9**		**ICD-10**	
**Myocardial infarction**	410, 412		I20.0, I21-I24	
**Cerebrovascular disease (including both ischemic stroke and cerebral hemorrhage)**	430-436		G45-G46, I60-I64	

© 2024 Gerber P et al. *JAMA Network Open.*

Confounders: In addition to matching, we will adjust for the following factors.

ICD-codes are listed in Table 5. Indexing occurs at study entry, and the search for covariates codes is limited to maximum 2 years before study entry.

- Diabetes: Yes or no.
- Hypertension: Yes or no.
- Peripheral vascular disease: Yes or no.

- Chronic obstructive pulmonary disease: Yes or no.
- Renal disease: Yes or no.
- Frailty, approximated by (three separate variables):
    - Deep vein thrombosis or pulmonary embolism: Yes or no.
    - Pneumonia: Yes or no.
    - Number of hospital admissions prior to cohort entry: Continuous.

Table 5. ICD-codes for comorbidities in Sweden.

	**ICD 9**	**ICD 10**
**Diabetes**	250	E11-E14
**Hypertension**	401-405	I10-I15
**Peripheral vascular disease**	440–447, V43E, 785E	I70–I73, I770–I771, K551, K558–K559, R02, Z958–Z959
**Chronic obstructive pulmonary disease, emphysema, bronchitis, bronchiectasis (smoking related lung disorders)**	466, 490-492, 494, 496	J40-J41, J43-J44, J47
**Renal disease**	403– 404, 580–588, V42A, V45B	I12–I13, N01, N03, N05, N07–N08, N171–N172, N18, N19, N25, Z49, Z940, Z992
**Deep vein thrombosis or pulmonary embolism**	415B, 416W, 451B	I802, I26
**Pneumonia**	480-486	J12-18

© 2024 Gerber P et al. *JAMA Network Open.*

Statistical analysis:

Study individuals enter upon date of bariatric surgery with matched controls entering on the same date. Follow-up will be divided at exactly one year after entry to account for latency in the first year. Comparison will be within the first year in exposed and non-exposed and within follow-up after one year between exposed and non-exposed. For the main outcome, the follow-up ends at the date of cancer, death, or end of the study period, whichever occurs first. For the secondary outcome, the follow-up ends upon cardio- or cerebrovascular disease, death, or end of the study period, whichever occurs first.

The crude cumulative incidence of the outcomes will be presented graphically. We will descriptively present 90-day mortality for operated patients. We will use multivariable Cox regression analysis to calculate hazard ratios with 95% confidence intervals. The hazard ratios will be adjusted for the confounders listed and categorized above. Stratified analyses on follow-up after the first year will be performed for country, age (e.g., 60-65, 66-70, >70 years based on distribution), sex (men and women), diabetes (yes or no) and duration of follow-up (categorized depending on the distribution of follow-up, e.g., 1-5 years, 6-10 years, 11-15 years, and >15 years).

A sub-analysis will be limited to patients operated with gastric bypass with matched controls.

Time plan:

Study protocol	Winter 2023/24
Data collection	Completed
Statistical analysis	Winter 2023/24
Manuscript drafting	Winter 2023/24
Submission	Spring 2024

eREFERENCES

1. Flegal KM, Kruszon-Moran D, Carroll MD, et al. Trends in Obesity Among Adults in the United States, 2005 to 2014. JAMA 2016;315:2284-91.
2. Haslam DW, James WP. Obesity. Lancet 2005;366:1197-209.
3. Prospective Studies C, Whitlock G, Lewington S, et al. Body-mass index and cause-specific mortality in 900 000 adults: collaborative analyses of 57 prospective studies. Lancet 2009;373:1083-96.
4. Kauppila JH, Tao W, Santoni G, et al. Effects of Obesity Surgery on Overall and Disease-Specific Mortality in a 5-Country Population-Based Study. Gastroenterology 2019;157:119-127 e1.
5. Reges O, Greenland P, Dicker D, et al. Association of Bariatric Surgery Using Laparoscopic Banding, Roux-en-Y Gastric Bypass, or Laparoscopic Sleeve Gastrectomy vs Usual Care Obesity Management With All-Cause Mortality. JAMA 2018;319:279-290.
6. Flum DR, Dellinger EP. Impact of gastric bypas operation on survival: a population-based analysis. Journal of the American College of Surgeons 2004;199:543-551.

© 2024 Gerber P et al. *JAMA Network Open.*

7. Adams TD, Gress RE, Smith SC, et al. Long-term mortality after gastric bypass surgery. N Engl J Med 2007;357:753-61.
8. Sjostrom L, Narbro K, Sjostrom CD, et al. Effects of bariatric surgery on mortality in Swedish obese subjects. N Engl J Med 2007;357:741-52.
9. Chang SH, Stoll CR, Song J, et al. The effectiveness and risks of bariatric surgery: an updated systematic review and meta-analysis, 2003-2012. JAMA Surg 2014;149:275-87.
10. Arterburn DE, Olsen MK, Smith VA, et al. Association between bariatric surgery and long-term survival. JAMA 2015;313:62-70.
11. Sjostrom L, Peltonen M, Jacobson P, et al. Bariatric surgery and long-term cardiovascular events. JAMA 2012;307:56-65.
12. Benotti P, Wood GC, Winegar DA, et al. Risk factors associated with mortality after Roux-en-Y gastric bypass surgery. Ann Surg 2014;259:123-30.
13. Koh CY, Inaba CS, Sujatha-Bhaskar S, et al. Outcomes of Laparoscopic Bariatric Surgery in the Elderly Population. Am Surg 2018;84:1600-1603.

© 2024 Gerber P et al. *JAMA Network Open.*
