# Supplementary material for: Incidence of Cancer and Cardiovascular Disease After Bariatric Surgery in Older Patients
Source: JAMA Netw Open. 2024 Aug 13;7(8):e2427457. doi: 10.1001/jamanetworkopen.2024.27457 (PMC11322843; doi:10.1001/jamanetworkopen.2024.27457)
Supplement: Supplement 2. — Data Sharing Statement [file jamanetwopen-e2427457-s002.pdf]

Data Sharing Statement

Gerber. Incidence of Cancer and Cardiovascular Disease After Bariatric Surgery in Older Patients. *JAMA Netw Open*. Published August 13, 2024.
doi:10.1001/jamanetworkopen.2024.27457

Data
Data available: No

Additional Information
Explanation for why data not available: Data may be obtained from a third party and are not publicly available.
